# Coal Use, Stove Improvement, and Adult Pneumonia Mortality in Xuanwei, China: A Retrospective Cohort Study

**DOI:** 10.1289/ehp.11521

**Published:** 2008-09-19

**Authors:** Min Shen, Robert S. Chapman, Roel Vermeulen, Linwei Tian, Tongzhang Zheng, Bingshu E. Chen, Eric A. Engels, Xingzhou He, Aaron Blair, Qing Lan

**Affiliations:** 1 Division of Cancer Epidemiology and Genetics, National Cancer Institute, National Institutes of Health, U.S. Department of Health and Human Services, Bethesda, Maryland, USA; 2 College of Public Health Sciences, Chulalongkorn University, Bangkok, Thailand; 3 Institute for Risk Assessment Sciences, Utrecht University, Utrecht, the Netherlands; 4 Stanley Ho Center for Emerging Infectious Diseases, School of Public Health, Chinese University of Hong Kong, Hong Kong Special Administrative Region, China; 5 Department of Epidemiology and Public Health, Yale University School of Medicine, New Haven, Connecticut, USA; 6 Department of Mathematics and Statistics, Concordia University, Montreal, Quebec, Canada; 7 Institute of Environmental Health and Engineering, Chinese Center for Disease Control and Prevention, Beijing, China

**Keywords:** coal, cohort study, indoor air pollution, pneumonia

## Abstract

**Background:**

In Xuanwei County, China, unvented indoor coal burning is strongly associated with increased risk of lung cancer and chronic obstructive pulmonary disease. However, the impact of coal burning and stove improvement on risk of pneumonia is not clear.

**Methods:**

We conducted a retrospective cohort study among all farmers born 1917 through 1951 and living in Xuanwei as of 1 January 1976. The analysis included a total of 42,422 cohort members. Follow-up identified all deaths in the cohort from 1976 through 1996. Ages at entry into and at exit from follow-up ranged from 24 to 59 years and from 25 to 80 years, respectively. The record search detected 225 deaths from pneumonia, and 32,332 (76%) were alive as of 31 December 1996. We constructed multivariable Cox models (time variable = age) to estimate hazard ratios (HRs) and 95% confidence intervals (CIs).

**Results:**

Use of coal, especially smokeless coal, was positively associated with pneumonia mortality. Annual tonnage and lifetime duration of smoky and smokeless coal use were positively associated with pneumonia mortality. Stove improvement was associated with a 50% reduction in pneumonia deaths (smoky coal users: HR, 0.521; 95% CI, 0.340–0.798; smokeless coal users: HR, 0.449; 95% CI, 0.215–0.937).

**Conclusions:**

Our analysis is the first to suggest that indoor air pollution from unvented coal burning is an important risk factor for pneumonia death in adults and that improving ventilation by installing a chimney is an effective measure to decrease it.

Nearly half of the world’s households use unprocessed biomass fuels and coal for cooking and heating (Smith et al. 2000). This is a major source of indoor air pollution and can increase the incidence of respiratory infection in both children and adults (Chen et al. 1990; Kilabuko et al. 2007; Mishra and Retherford 1997; Sharma et al. 1998; Sumer et al. 2004). Death due to acute respiratory infection (ARI) is the leading burden of death in the world (8.5%), especially in developing countries (9.4%) (Murray and Lopez 1996). Acute lower respiratory tract infection (ALRI) is much more serious than upper respiratory tract infection, and pneumonia is the most serious form of ALRI.

Xuanwei County is well known for its high incidence of lung cancer, attributed mainly to the wide use of smoky coal mined locally and the use of unvented fire-pit stoves (Mumford et al. 1987). It accounts for more than 90% of lung cancer cases for both men and women (He and Yang 1994).

To control the high incidence of lung cancer, the Xuanwei County government implemented a program in the late 1970s to change the traditional unvented fire-pit stove to a stove with chimney to improve ventilation and reduce exposures. To quantitatively assess the effect of coal use and stove improvement on morbidity and mortality in adults, we conducted a retrospective cohort study in Xuanwei County from 1992 to 1996. We previously reported that the installation of chimneys was strongly associated with reduced risk of incident lung cancer and chronic obstructive pulmonary disease (COPD) (Chapman et al. 2005; Lan et al. 2002). Previous studies suggested indoor and outdoor air pollution may increase the morbidity and mortality of childhood pneumonia (Chen et al. 1990; Dherani et al. 2008; Nyberg and Pershagen 2000; Smith et al. 2000). However, the association between indoor air pollution due to coal use and pneumonia mortality in adults has not been reported in a case–control or cohort study. Here we report the analysis of the role of household fuel use, stove improvement, and occupational exposures on pneumonia mortality in this cohort.

## Materials and Methods

### Cohort study design and data collection

In 1992, we searched local administrative records to identify all farmers born from 1917 through 1951 and living in four of Xuanwei’s communes (Rong Cheng, Lai Bin, Jing Wai, and Re Shui) as of 1 January 1976. In total, we identified 44,580 records. A total of 42,422 farmers were available for study after excluding 2,108 subjects who had moved out of the study area and 50 subjects with insufficient information for analysis.

In three of the four communes, most residents used smoky coal (mainly from Lai Bin). In the other commune, most residents used smokeless coal (mainly from Re Shui). In Xuanwei, smoky and smokeless coals correspond to bituminous coal and anthracite, respectively. The terms “smoky” and “smokeless” coal have been used in many published reports from studies in this area (Chapman et al. 2005; Lan et al. 2002; Mumford et al. 1987), and the use of these terms has been broadly accepted. The coal in this region likely has distinct characteristics, which are still being evaluated, so we use the terms “smoky” and “smokeless” coal here in the context of Xuanwei. The physiochemical characteristics of the two types of coal are different in Xuanwei. Smokeless coal contains more sulfur (1.7% vs. 0.25%), based on an earlier elemental analysis, and less total suspended particulates (1.59 vs. 5.64) than does smoky coal (He and Yang 1994; Mumford et al. 1987).

In 1992, trained interviewers administered a standardized questionnaire for all cohort members. They interviewed subjects directly, when feasible, or surrogate respondents for subjects deceased or not present (41%). This study was approved by the Institutional Review Board of the Chinese Academy of Preventive Medicine. Interviewers obtained written informed consent from all literate questionnaire respondents and verbal consent from others. The questionnaire elicited information on demographic factors, residential history, lifetime use of household stove and fuel type, occupational history, tobacco smoking, cooking food, time spent indoors and outdoors, and medical history. The questionnaire interview typically took about 40 min.

We identified deaths among these subjects from 1976 through 1996 using information from hospitals, local public security bureaus, and local public health bureaus. All cohort members lived within 10–12 miles of one of the four large hospitals in Xuanwei. We also searched records of the Qujing District Hospital (Qujing is the administrative region that includes Xuanwei County) and the Yunnan Province Hospital to identify patients who died outside Xuanwei. The diagnosis of pneumonia during the time period of cohort follow-up was based primarily on symptoms, clinical exam, and a chest X-ray. Empirical treatment with antibiotics was used in general. We excluded tuberculosis and COPD when pneumonia was diagnosed as the cause of death. If a patient died from pneumonia, a physician completed a death certificate, which is how death from pneumonia in Xuanwei is documented. A death certificate is required for official documentation in hospitals, public security bureaus, and public health bureaus. Information abstracted from the death records included name, sex, date of birth, residence, date of death, and cause of death. We used name, sex, date of birth, and residence information to identify unique study subjects in the cohort. We included a total of 787,814 person-years during follow-up, with an average of 18.6 person-years per subject. We coded the cause of death using the *International Classification of Diseases, 9th Revision* (ICD-9; World Health Organization 1975).

### Statistical analysis

We included 42,422 subjects (21,701 men and 20,721 women) in the analysis. For each subject, we calculated the age at entry into cohort follow-up as the number of years from the subject’s birth date to 1 January 1976. After entry, subjects could exit from follow-up in one of the following three ways: death from pneumonia, death from other causes before the study ended in 1996, or living as of 31 December 1996. Ages at entry into and at exit from follow-up ranged from 24 to 59 years and from 25 to 80 years, respectively.

In this report, the term “stove improvement” refers to changing permanently from fire-pit or stove without a chimney to stove with a chimney. We constructed multivariable Cox models using the SAS PHREG procedure (version 9.1.3; SAS Institute Inc., Cary, NC, USA) to estimate hazard ratios (HRs) and 95% confidence intervals (CIs). The time variable (time axis) was age, starting with age as of 1 January 1976 and ending with age at exit from follow-up. The outcome event was death from pneumonia (ICD-9 codes 480, 481, 482, 483, 485, and 486). We considered individuals who died from other causes before the study ended in 1996, or who remained alive as of 31 December 1996, as censored (competing-risks approach). In the models, we assessed the effects of coal use, stove improvement, years of smoking, and years of cooking as time-dependent continuous or categorical variables based on the starting age for each of these activities. To adjust for potential birth cohort effects, models were stratified into seven birth cohort strata: 1917–1921, 1922–1926, 1927–1931, 1932–1936, 1937–1941, 1942–1946, and 1947–1951.

We analyzed the data in four Cox models: two separate models for men and women, and two separate models for smoky coal and smokeless coal users. Nearly all subjects used either smoky or smokeless coal, so we included only the variable “use of smokeless coal” (yes or no) in the sex-specific models. We calculated the time-dependent variables tonnage per year and duration in years for all coals combined and for smoky coal and smokeless coal separately, categorized into tertiles. We used two time-dependent variables for stove improvement: a binary variable for the overall effect of stove improvement (0 before and 1 after the age at stove improvement) and a categorical variable indicating time since stove improvement (0–6, 7–13, ≥ 14 years). We analyzed the time-dependent variable for duration of tobacco smoking (20–40 years or ≥ 40 years), relative to a reference category of subjects who had not smoked or smoked < 20 years, in the men’s model. We used another time-dependent binary variable of tobacco smoking in coal type–specific models, which switched from 0 to 1 at the age at first smoking. Similarly, we used a time-dependent binary variable for cooking (yes/no) in the men’s model and coal type–specific models; we included two time-dependent variables for different durations (20–40 years or ≥ 40 years) of cooking food, relative to a reference category of subjects who had not cooked food or cooked for < 20 years, in the women’s model.

In addition to the variables mentioned above, we included the following variables in the Cox models: *a*) sex in the coal type–specific models; *b*) having any education, to adjust for socioeconomic status; *c*) average number of rooms in the home over the lifetime, to adjust for socioeconomic status and for potential effects of coal smoke dilution in the home; *d*) average number of people in the home over the lifetime, to adjust for crowding pattern; *e*) spending ≥ 7 daily waking hours indoors through 20 years of age, to adjust for elevated exposure to indoor coal smoke early in life; *f*) reporting diagnosed chronic bronchitis, emphysema, or asthma (often observed to be pneumonia risk factors); and *g*) a dummy variable for ever being a coal miner in the men’s model (originally, occupational history also included being a driver or a construction worker, but no pneumonia deaths occurred among these groups).

## Results

Table 1 shows characteristics of the 42,422 subjects included in data analyses. Most people used either smoky coal or smokeless coal as the major fuel type during their lifetime. Only 336 subjects ever used both smoky coal and smokeless coal, and 263 subjects used neither of them.

Lung cancer was the major cause of death with 2,459 deaths during follow-up. There were 225 deaths from pneumonia. The lung cancer mortality rates in the smoky coal–using communes were about 17 times higher in men and 39 times higher in women compared with national rates in China (Mumford et al. 1987). This is the region where we established our smoky coal–using cohort. As a consequence, the ratio of mortality from lung cancer to mortality from pneumonia is far higher in men and women in this region than in other parts of China and in the world.

The mean age at death due to pneumonia was 63.2 years. When the follow-up ended in 1996, 32,332 (76%) subjects were alive. Age-adjusted mortality rates of pneumonia were higher in smokeless coal users (43.8 vs. 27.8 per 100,000) and among people who had not improved their stove (48.9 vs. 22.4 per 100,000). Survival rates were lower among smoky coal users compared with smokeless coal users (74% vs. 81%) and among people living in houses without chimneys than among those in houses with chimneys (70% vs. 80%).

About half of men (46%), but only 23% of women, had some education. About 5% and 6% of men had been coal miners and construction workers, respectively. More people who used smoky coal and experienced stove improvement had a nonfarming job.

Men spent less time in the home than did women. About 10% of subjects had been diagnosed with chronic bronchitis, asthma, or emphysema. Most men (92%) had smoked, and few had quit; few women smoked (< 1%). Almost all women (98%) cooked food for the family from early in life, and only 8% of men ever cooked.

Seventy percent of people used smoky coal. Most families burned about 3 tons of coal each year. Around 60% of families improved stoves with chimneys before follow-up ended. More people using smoky coal improved their stoves than did people using smokeless coal (75% vs. 19%) because the government sponsored a stove improvement campaign in smoky coal–using areas in the 1970s to control the epidemic of lung cancer.

Table 2 summarizes the Cox regression models for pneumonia mortality in men and women. Stove improvement was an important protective factor (HR: men, 0.493; 95% CI, 0.312–0.779; women, 0.534; 95% CI, 0.323–0.882). When we categorized duration of stove improvement, the protection was comparable across different periods among men but weaker among women with longer duration (HRs of 0.243, 0.513, and 1.053, respectively). However, we found no statistical interaction between stove improvement and sex. Using smokeless coal was associated with a 50% higher risk of pneumonia death in both men and women. When we combined men and women in one model, the association was highly significant (HR for using smokeless coal, 1.580; 95% CI, 1.147–2.174; *p* = 0.005) (data not shown). The annual tonnage of coal used and the duration of coal use in years were associated with significantly increased risk at the highest tertiles. The HRs for tonnage and duration of coal use were a bit larger among women than among men. The number of people in the home was associated with a borderline increase of risk of pneumonia death in women (HR, 1.180; 95% CI, 0.994–1.401). History of non-malignant pulmonary diseases was a major risk factor for pneumonia death (HR: men, 2.009; 95% CI, 1.316–3.066; women, 2.126; 95% CI, 1.347–3.356). Smoking for ≥ 40 years among men and cooking for ≥ 20 years among women were associated with a nonsignificantly increased risk of pneumonia death. Educational attainment was associated with a reduced risk of pneumonia death in men only (HR, 0.502; 95% CI, 0.287–0.876).

In the model for smoky coal users (Table 3), the protective role of stove improvement was strong (HR, 0.521; 95% CI, 0.340–0.798). The highest categories of annual smoky coal use and duration were associated with approximately 2-fold (HR, 1.802; 95% CI, 1.089–2.982) and 3-fold (HR, 3.098; 95% CI, 0.939–10.219) risks of pneumonia death, respectively. We found no clear difference in amount of protection according to different periods of time after stove improvement. The number of rooms and number of people in the home were associated with altered risk of pneumonia death (HR: for every additional room, 0.618; 95% CI, 0.437–0.872; for every additional person, 1.334; 95% CI, 1.132–1.573). Finally, people with any education had lower risk of pneumonia death than did people without education (HR, 0.452; 95% CI, 0.231–0.882).

In the model for smokeless coal users (Table 3), higher annual coal consumption (HR for ≥ 3.3 vs. < 3 tons, 1.671; 95% CI, 0.984–2.837) and longer duration of use (HR: for 43 to 56 vs. < 43 years, 3.210; 95% CI, 1.213–8.490; for ≥ 56 vs. < 43 years, 2.613; 95% CI, 1.178–5.798) were significantly associated with higher risk of death from pneumonia. The protective role of stove improvement was strong (HR, 0.449; 95% CI, 0.215–0.937). Nonmalignant pulmonary disease history was associated with a large increased risk of pneumonia death only in smokeless coal users (HR, 3.660; 95% CI, 2.449–5.471). In addition, staying at home for a longer time was associated with an elevated risk of death from pneumonia (HR for ≥ 7 hr/day vs. < 7 hr, 1.524; 95% CI, 1.010–2.298).

## Discussion

In the analysis, we found that use of coal, especially smokeless coal, and stove improvement that decreases coal smoke level indoors were important risk and protective factors, respectively, for pneumonia death in Xuanwei. The fatality rate of pneumonia would be very high if patients were not treated immediately with antibiotics, which often occurred in rural areas of China. Many studies have found that solid fuel use was associated with high incidence of ARIs in children (Mahalanabis et al. 2002; Smith et al. 2000). Smith et al. (2000) reviewed studies conducted in developing countries about effects of indoor air pollution on ALRI, including pneumonia in children, and concluded that indoor air pollutants were associated with increased risk of ALRI. Many investigators also reported that air pollution levels, due to coal burning or other urban air pollutants, were positively associated with the hospital emergency visits for pneumonia (Fusco et al. 2001; Ozdilek 2006; Peel et al. 2005; Wong et al. 1999). Our study provides evidence for the first time that indoor air pollution due to solid fuel burning increases pneumonia deaths in adults. It also raises suspicion that indoor coal burning is associated with pneumonia risk in children.

Coal smoke contains many chemicals (e.g., nitrogen oxides, sulfur oxides, ozone, carbon monoxide) and particles that irritate respiratory tracts and lung, adversely affect the host’s defense systems against pathogens, and elevate the risk of respiratory tract infections (Smith et al. 2000). Coal smoke may also increase the severity of respiratory infections by causing inflammation in pulmonary airways (American Thoracic Society 1996). Because of the long-term high indoor air pollution pattern in Xuanwei, the prevalence of chronic respiratory tract inflammation is thought to be high because of the high incidence of COPD (Zhou et al. 1995). Consequently, pneumonia could plausibly lead to a more severe outcome (i.e., death).

Our analysis demonstrated that stove improvement benefits not only lung cancer and COPD (Chapman et al. 2005; Lan et al. 2002), but also pneumonia (present results). It provides similar health benefits in homes using either smoky coal or smokeless coal. This effect would have remained statistically significant even if there had been a large unadjusted design effect in the data. In an intervention study in smoky coal users in this region, using an improved stove reduced the levels of sulfur dioxide from 0.32 to 0.02 mg/m^3^ and total suspended particulate matter (PM) from 5.64 to 1.21 mg/m^3^, based on a 5-day measurement (He and Yang 1994). In 1995, an indoor air pollution study simulated pollutant concentrations in the homes of a small group of cohort members with and without stove improvement to assess changes in pollutant concentrations. Average concentrations of particulate matter with aerodynamic diameter ≤ 10 μm (PM_10_) with unvented burning of coal were almost three times higher than for burning with open chimneys (2.08 vs. 0.71 mg/m^3^) (Lan et al. 2002). In addition, a small study in two homes of smokeless coal users showed a nonsignificant mean (± SD) decrease of PM_10_ from 0.76 ± 0.30 to 0.62 ± 0.17 mg/m^3^ after stove improvement (Tian L, unpublished data). Another study in Burkina Faso (Africa) found that good ventilation conditions considerably reduced the fraction of child ARI attributable to smoke from biomass solid fuel (Akunne et al. 2006). Ezzati and Kammen (2001) reported that exposure to PM_10_ particulates was associated with ARI proportionally in people who burned biomass fuel.

We found that, among women, protection was stronger soon after stove improvement and vanished in later periods. This may be attributable to decreased effectiveness of ventilation with passage of time after improvement. Our researchers observed that some families could not maintain the stove in good condition. However, we did not find a significant interaction between stove improvement and sex. Such heterogeneity of risk reduction between sexes may be due to statistical variation.

Unlike lung cancer, for which smoky coal use was the dominant risk factor (Mumford et al. 1987), in the present study the smokeless coal was associated with a higher risk of pneumonia death than smoky coal. The nitrogen content of these coals is not known. However, smokeless coal contains more sulfur than does smoky coal (He and Yang 1994; Mumford et al. 1987). Therefore, burning smokeless coal likely generated more airborne sulfur oxides, which have been identified as risk factors for pneumonia (American Thoracic Society 1996; Oehme et al. 1996).

Coal emissions have additional components that can irritate the respiratory system and plausibly increase the risk of pneumonia death, including ozone, inhalable particles, heavy metals, and dioxins. We found the HRs were proportional to the amount and the duration of coal used per family for both smoky coal and smokeless coal. The observed exposure–response associations lend weight to causal inference.

Occurrence of chronic nonmalignant pulmonary diseases, an acknowledged risk factor for pneumonia, was a strong risk factor for pneumonia death in smokeless coal users. The occurrence of these pulmonary diseases increased in Xuanwei because of indoor air pollution (Chapman et al. 2005). It also may be on the causal pathway from coal use to pneumonia death. Therefore, the model may be overadjusted somewhat. However, such overadjustment had no substantial impact on the modeled HRs and CIs for coal-related variables compared with models excluding these variables. In addition, after excluding the subjects with a history of COPD or tuberculosis, the effects of stove improvement and coal use remained unchanged.

We found a nonsignificant effect for smoking among men and for cooking among women on pneumonia mortality in Xuanwei, which may reflect the strong effect of coal use, as previously observed for lung cancer (He and Yang 1994). It may also be attributable to the small case number and the consequent limited power to detect associations in our study. Power to test associations of occupation with pneumonia risk was also limited.

Compared with previous reports from this cohort study (Chapman et al. 2005; Lan et al. 2002), the present analysis included a commune where most people used smokeless coal. Such inclusion made it possible to compare the effect of different coals on pneumonia risk. One limitation of the study is the lack of detailed exposure assessment data for households before and after stove improvement using smoky coal or smokeless coal, even though a few small-scale studies in this area provided some basic data (He and Yang 1994). Lack of detailed exposure assessment limits comparability of our study with other studies. We are conducting an extensive exposure assessment study in this area to explore the pollution of different coals in different settings, which includes long-term stationary and personal exposure assessment and detailed measurement of different exposures. Those results will be available at a later date; the results in the present stand alone and are important for understanding the risk of pneumonia. The exposure assessment study, once complete, will further inform the interpretation of results reported in the present article.

There is a possibility of misclassification of the diagnosis of pneumonia with other respiratory diseases (e.g., tuberculosis, bronchitis). In addition, we cannot exclude the possibility that some pneumonia cases may also have had undiagnosed lung cancer. However, pneumonia diagnosis was made by government hospitals and was mainly based on symptoms, clinical exam, and a chest X-ray. Doctors throughout Xuanwei County used the same procedures and criteria to diagnose pneumonia and document cause of death. If there is some minimal error in the diagnosis of pneumonia in this population, it is highly unlikely that the error would be related to whether or not a patient had a home with or without improved ventilation. Thus any error in diagnosis would not be expected to inflate the effect of improved ventilation.

In conclusion, our results strongly support the hypothesis that unvented household coal burning is an important risk factor for pneumonia death in adults. Stove improvement was followed by reduced risk of death from pneumonia in coal users. Indoor air pollution due to solid fuel burning is a serious health hazard in developing countries. Coal smoke exposure is associated with multiple medical conditions, mainly lung-related diseases. The population at risk for illness mediated by indoor air pollution numbers billions worldwide (World Health Organization 1997). There were 1.6 million of excess deaths due to indoor air pollution (Smith et al. 2004). If possible, cleaner fuel including processed solid fuels shall be used to reduce generation of airborne pollutants. Also, installation of chimneys and other measures should be implemented to improve ventilation and thereby reduce indoor pollution levels and risk of adverse health outcomes.

## Figures and Tables

**Table 1:** Characteristics of cohort members included in the analysis, Xuanwei, China.

Variable	Men	Women	Smoky coal users	Smokeless coal users	With stove improvement	No stove improvement	All
Total included in data analysis (no.)	21,701	20,721	29,890	12,605	24,715	17,707	42,422
Person-years (no.)	401,985	385,829	550,611	240,288	478,340	309,474	787,814
Age as of 1 January 1976 (mean ± SD)	39.5 ± 10.6	39.0 ± 10.6	38.9 ± 10.5	39.9 ± 10.8	38.4 ± 10.2	40.4 ± 11.0	39.2 ± 10.6
Age at exit from follow-up (mean ± SD)	58.0 ± 9.8	57.6 ± 9.7	57.3 ± 9.6	59.0 ± 10.0	57.8 ± 9.5	57.9 ± 10.1	57.8 ± 9.8
Pneumonia death, 1976–1996 [no. (%)]	125 (0.6)	100 (0.5)	108 (0.4)	114 (0.9)	69 (0.3)	156 (0.9)	225 (0.5)
Age at pneumonia death (mean ± SD)	63.4 ± 8.9	62.9 ± 9.3	62.1 ± 10.1	64.3 ± 8.2	64.1 ± 9.8	62.8 ± 8.7	63.2 ± 9.0
Age-adjusted mortality (no. per 100,000 population)^a^	35.8	31.2	27.8	43.8	22.4	48.9	33.6
Died without pneumonia during follow-up [no. (%)]	5,182 (24)	4,683 (23)	7,510 (25)	2,254 (18)	4,793 (19)	5,072 (29)	9,865 (23)
Alive at the end of follow-up in 1996 [no. (%)]	16,394 (76)	15,938 (77)	22,272 (74)	10,237 (81)	19,853 (80)	12,479 (70)	32,332 (76)
Commune [no. (%)]							
Rong Cheng	5,705 (26)	6,064 (29)	11,763 (39)	160 (1)	7,130 (29)	4,639 (26)	11,769 (28)
Jing Wai	1,003 (5)	943 (5)	1,920 (6)	41 (0.3)	1,667 (7)	279 (2)	1,946 (4)
Lai Bin	8,405 (39)	7,754 (37)	16,099 (54)	75 (0.6)	13,626 (55)	2,533 (14)	16,159 (38)
Re Shui	6,571 (30)	5,939 (29)	73 (0.2)	12,326 (98)	2,263 (9)	10,247 (58)	12,510 (29)
Other	17 (0.1)	21 (0.1)	35 (0.1)	3 (0.02)	29 (0.1)	9 (0.05)	38 (0.09)
With any education [no. (%)]	9,953 (46)	4,836 (23)	10,803 (36)	4,033 (32)	8,951 (36)	5,838 (33)	14,789 (35)
Any other work in addition to farming [no. (%)]	4,816 (22)	1,193 (6)	5,781 (19)	278 (2)	4,315 (17)	1,694 (10)	6,009 (14)
Coal miner	1,117 (5)	28 (0.1)	1,124 (4)	24 (0.2)	955 (4)	190 (1)	1,145 (3)
Driver	115 (0.5)	3 (0.01)	113 (0.4)	5 (0.04)	74 (0.3)	44 (0.2)	118 (0.3)
Construction	1,395 (6)	41 (0.2)	1,421 (5)	16 (0.1)	1,032 (4)	404 (2)	1,436 (3)
Others	2,189 (10)	1,121 (5)	3,123 (10)	233 (2)	2,254 (9)	1,056 (6)	3,310 (8)
Average number of people in home over lifetime (mean ± SD)	5.2 ± 1.2	5.3 ± 1.1	5.2 ± 1.2	5.4 ± 1.2	5.3 ± 1.2	5.3 ± 1.2	5.3 ± 1.2
Average number of rooms in home over lifetime (mean ± SD)	1.7 ± 0.8	1.7 ± 0.8	1.6 ± 0.8	2.0 ± 0.8	1.6 ± 0.8	1.9 ± 0.8	1.7 ± 0.8
People with ≥ 7 waking hours indoors per day through age 20 [no. (%)]	8,213 (38)	9,026 (44)	13,972 (47)	3,283 (26)	11,690 (47)	5,549 (31)	17,239 (41)
Chronic bronchitis, asthma, or emphysema history [no. (%)]	2,130 (10)	2,116 (10)	3,296 (11)	916 (7)	1,905 (8)	2,341 (13)	4,246 (10)
Ever smokers [no. (%)]	20,010 (92)	61 (0.3)	13,943 (47)	6,048 (48)	11,684 (47)	8,387 (47)	20,071 (47)
Age starting to smoke (mean ± SD)	21.1 ± 3.6^b^	22.6 ± 5.3	21.6 ± 3.9^c^	20.1 ± 2.7^d^	21.5 ± 3.8^e^	20.7 ± 3.4^f^	21.1 ± 3.6^g^
Years of smoking in smokers (mean ± SD)	33.8 ± 10.4^b^	34.4 ± 10.1	32.9 ± 10.2^c^	35.7 ± 10.4^d^	33.3 ± 10.3^e^	34.3 ± 10.5^f^	33.8 ± 10.4^g^
Ever quitting smoking [no. (%)]	262 (1)	3 (0.01)	232 (0.8)	32 (0.2)	174 (0.7)	91 (0.5)	265 (0.6)
Ever cooked food [no. (%)]	1,836 (8)	20,315 (98)	15,922 (53)	6,388 (51)	12,936 (52)	9,215 (52)	22,151 (52)
Age starting to cook (mean ± SD)	27.7 ± 14.7	18.3 ± 3.6	18.9 ± 6.4	19.6 ± 4.9	19.0 ± 6.3	19.2 ± 5.7	19.1 ± 6.1
Years of cooking (mean ± SD)	23.9 ± 13.9	33.9 ± 10.2	33.0 ± 11.0	33.3 ± 10.7	33.3 ± 10.9	32.9 ± 10.9	33.1 ± 10.9
People who ever used smoky coal [no. (%)]	15,114 (70)	14,776 (71)	29,890 (100)	336 (3)	22,405 (91)	7,485 (42)	29,890 (70)
Tons per year (mean ± SD)	3.3 ± 1.1	3.3 ± 1.0	3.3 ± 1.0	3.1 ± 1.4	3.4 ± 1.0	2.9 ± 1.0	3.3 ± 1.0
People who ever used smokeless coal [no. (%)]	6,497 (30)	6,108 (29)	336 (1)	12,605 (100)	2,447 (10)	10,158 (57)	12,605 (30)
Tons per year (mean ± SD)	3.0 ± 1.6	3.0 ± 1.6	3.1 ± 1.3	3.0 ± 1.6	2.8 ± 1.4	3.0 ± 1.6	3.0 ± 1.6
People with stove improvement [no. (%)]	12,529 (58)	12,186 (59)	22,405 (75)	2,447 (19)	24,715 (100)		24,715 (58)
Age at stove improvement (mean ± SD)	41.8 ± 11.2	41.0 ± 11.0	41.6 ± 10.8	39.6 ± 13.6	41.4 ± 11.1		41.4 ± 11.1
Time from stove improvement until exit from follow-up	16.3 ± 6.7	16.4 ± 6.7	16.0 ± 5.9	19.5 ± 11.3	16.4 ± 6.7		16.4 ± 6.7

Values are no., no. (%), or mean ± SD.

a Adjusted to the world standard population in 1976.

b Number of subjects with information = 19,995.

c Number of subjects with information = 13,930.

d Number of subjects with information = 6,046.

e Number of subjects with information = 11,676.

f Number of subjects with information = 8,380.

g Number of subjects with information = 20,056.

**Table 2:** Sex-specific Cox regression models of pneumonia death, Xuanwei, 1976–1996.

	Men		Women	
Variable	HR (95% CI)	*p*-Value	HR (95% CI)	*p*-Value
Stove improvement (binary)				
No	1		1	
Yes	0.493 (0.312–0.779)	0.002	0.534 (0.323–0.882)	0.014
Time since stove improvement (ordinal)				
No improvement	1		1	
< 7 years	0.442 (0.220–0.889)	0.022	0.243 (0.095–0.627)	0.003
≥ 7 years to < 14 years	0.542 (0.304–0.968)	0.038	0.513 (0.258–1.019)	0.057
≥ 14 years	0.480 (0.230–1.000)	0.050	1.053 (0.552–2.007)	0.876
Ever use smokeless coal				
No	1		1	
Yes	1.522 (0.983–2.355)	0.060	1.444 (0.899–2.319)	0.129
Tons of coal used per year^a^				
< 2.57	1		1	
≥ 2.57 to < 3.75	0.787 (0.502–1.234)	0.297	0.962 (0.573–1.614)	0.883
≥ 3.75	1.535 (0.981–2.404)	0.061	1.881 (1.141–3.101)	0.013
Years of coal use^a^				
< 45 years	1		1	
≥ 45 to < 57 years	1.094 (0.324–3.700)	0.885	2.334 (0.822–6.622)	0.111
≥ 57 years	2.482 (1.112–5.543)	0.027	2.668 (1.069–6.661)	0.035
Waking hours/day indoors through 20 years of age				
< 7 hr	1		1	
≥ 7 hr	0.896 (0.609–1.318)	0.577	1.237 (0.820–1.866)	0.311
No. of rooms in home				
Every additional room	0.825 (0.634–1.074)	0.152	1.006 (0.770–1.313)	0.967
No. of people in home				
Every additional person	1.095 (0.943–1.271)	0.234	1.180 (0.994–1.401)	0.058
Diagnosed chronic bronchitis, emphysema, or asthma				
No	1		1	
Yes	2.009 (1.316–3.066)	0.001	2.126 (1.347–3.356)	0.001
Cooking (ordinal)				
< 20 years	—	—	1	
≥ 20 to < 40 years	—	—	1.740 (0.275–10.990)	0.556
≥ 40 years	—	—	2.911 (0.467–18.131)	0.252
Cooking (binary)				
No	1	—	—	—
Yes	0.705 (0.335–1.483)	0.357	—	—
Work as coal miner				
No	1		—	—
Yes	1.022 (0.318–3.281)	0.971	—	—
Tobacco smoking				
< 20 years	1		—	—
≥ 20 to < 40 years	0.935 (0.393–2.227)	0.879	—	—
≥ 40 years	1.639 (0.702–3.826)	0.254	—	—
Education				
No education	1		1	
With education	0.502 (0.287–0.876)	0.015	1.959 (0.959–4.001)	0.065

a Smoky and smokeless coal types combined.

**Table 3:** Fuel type–specific Cox regression models of pneumonia death, Xuanwei, 1976–1996.

	Smoky coal users		Smokeless coal users	
Variable	HR (95% CI)	*p*-Value	HR (95% CI)	*p*-Value
Stove improvement (binary)				
No	1		1	
Yes	0.521 (0.340–0.798)	0.003	0.449 (0.215–0.937)	0.033
Time since stove improvement (ordinal)				
No improvement	1		1	
< 7 years	0.353 (0.187–0.667)	0.001	0.359 (0.087–1.477)	0.156
≥ 7 years to < 14 years	0.626 (0.366–1.069)	0.086	0.360 (0.088–1.477)	0.156
≥ 14 years	0.720 (0.382–1.356)	0.309	0.596 (0.216–1.643)	0.317
Tons of smoky coal used per year				
< 3	1		—	—
≥ 3 to < 4	1.627 (0.997–2.657)	0.052	—	—
≥ 4	1.802 (1.089–2.982)	0.022	—	—
Tons of smokeless coal used per year				
< 2	—	—	1	
≥ 2 to < 3.3	—	—	0.746 (0.436–1.278)	0.287
≥ 3.3	—	—	1.671 (0.984–2.837)	0.058
Years of smoky coal use				
< 45 years	1		—	—
≥ 45 to < 56 years	2.043 (0.534–7.810)	0.297	—	—
≥ 56 years	3.098 (0.939–10.219)	0.063	—	—
Years of smokeless coal use				
< 43 years	—	—	1	
≥ 43 to < 56 years	—	—	3.210 (1.213–8.490)	0.019
≥ 56 years	—	—	2.613 (1.178–5.798)	0.018
Waking hours/day indoors through age 20				
< 7 hr	1		1	
≥ 7 hr	0.714 (0.482–1.058)	0.093	1.524 (1.010–2.298)	0.045
No. of rooms in home				
Every additional room	0.618 (0.437–0.872)	0.006	1.133 (0.930–1.380)	0.215
No. of people in home				
Every additional person	1.334 (1.132–1.573)	0.001	0.887 (0.759–1.037)	0.133
Diagnosed chronic bronchitis, emphysema, or asthma				
No	1		1	
Yes	0.852 (0.476–1.523)	0.588	3.660 (2.449–5.471)	< 0.0001
Tobacco smoking				
No	1		1	
Yes	0.569 (0.240–1.350)	0.201	1.348 (0.416–4.367)	0.619
Cooking				
No	1		1	
Yes	0.845 (0.371–1.928)	0.690	0.819 (0.314–2.137)	0.683
Sex				
Men	1		1	
Women	0.595 (0.223–1.589)	0.300	1.307 (0.356–4.800)	0.686
Education				
No education	1		1	
With education	0.452 (0.231–0.882)	0.020	1.412 (0.801–2.492)	0.233

## References

[b1-ehp-117-261] Akunne AF, Louis VR, Sanon M, Sauerborn R (2006). Biomass solid fuel and acute respiratory infections: the ventilation factor. Int J Hyg Environ Health.

[b2-ehp-117-261] American Thoracic Society, Committee of the Environmental and Occupational Health Assembly (1996). Health effects of outdoor air pollution. Am J Respir Crit Care Med.

[b3-ehp-117-261] Chapman RS, He X, Blair AE, Lan Q (2005). Improvement in household stoves and risk of chronic obstructive pulmonary disease in Xuanwei, China: retrospective cohort study. BMJ.

[b4-ehp-117-261] Chen BH, Hong CJ, Pandey MR, Smith KR (1990). Indoor air pollution in developing countries. World Health Stat Q.

[b5-ehp-117-261] Dherani M, Pope D, Mascarenhas M, Smith KR, Weber M, Bruce N (2008). Indoor air pollution from unprocessed solid fuel use and pneumonia risk in children aged under five years: a systematic review and meta-analysis. Bull WHO.

[b6-ehp-117-261] Ezzati M, Kammen D (2001). Indoor air pollution from biomass combustion and acute respiratory infections in Kenya: an exposure-response study. Lancet.

[b7-ehp-117-261] Fusco D, Forastiere F, Michelozzi P, Spadea T, Ostro B, Arca M (2001). Air pollution and hospital admissions for respiratory conditions in Rome, Italy. Eur Respir J.

[b8-ehp-117-261] He X, Yang R (1994). Lung Cancer and Indoor Air Pollution from Coal Burning [in Chinese].

[b9-ehp-117-261] Kilabuko JH, Matsuki H, Nakai S (2007). Air quality and acute respiratory illness in biomass fuel using homes in Bagamoyo, Tanzania. Int J Environ Res Public Health.

[b10-ehp-117-261] Lan Q, Chapman RS, Schreinemachers DM, Tian L, He X (2002). Household stove improvement and risk of lung cancer in Xuanwei, China. J Natl Cancer Inst.

[b11-ehp-117-261] Mahalanabis D, Gupta S, Paul D, Gupta A, Lahiri M, Khaled MA (2002). Risk factors for pneumonia in infants and young children and the role of solid fuel for cooking: a case-control study. Epidemiol Infect.

[b12-ehp-117-261] Mishra V, Retherford RD (1997). Cooking smoke increases the risk of acute respiratory infection in children. Natl Fam Health Surv Bull.

[b13-ehp-117-261] Mumford JL, He XZ, Chapman RS, Cao SR, Harris DB, Li XM (1987). Lung cancer and indoor air pollution in Xuan Wei, China. Science.

[b14-ehp-117-261] Murray CJL, Lopez AD (1996). Global Burden of Disease.

[b15-ehp-117-261] Nyberg F, Pershagen G (2000). Epidemiologic studies on the health effects of ambient particulate air pollution. Scand J Work Environ Health.

[b16-ehp-117-261] Oehme FW, Coppock RW, Mostrom MS, Khan AA (1996). A review of the toxicology of air pollutants: toxicology of chemical mixtures. Vet Hum Toxicol.

[b17-ehp-117-261] Ozdilek HG (2006). An analogy on assessment of urban air pollution in Turkey over the turn of the millennium (1992–2001). Environ Monit Assess.

[b18-ehp-117-261] Peel JL, Tolbert PE, Klein M, Metzger KB, Flanders WD, Todd K (2005). Ambient air pollution and respiratory emergency department visits. Epidemiology.

[b19-ehp-117-261] Sharma S, Sethi GR, Rohtagi A, Chaudhary A, Shankar R, Bapna JS (1998). Indoor air quality and acute lower respiratory infection in Indian urban slums. Environ Health Perspect.

[b20-ehp-117-261] Smith KR, Mehta S, Feuz M, Ezzati M, Rodgers AD, Lopez AD, Murray CJL (2004). Indoor smoke from household solid fuels. Comparative Quantification of Health Risks: Global and Regional Burden of Disease due to Selected Major Risk Factors.

[b21-ehp-117-261] Smith KR, Samet JM, Romieu I, Bruce N (2000). Indoor air pollution in developing countries and acute lower respiratory infections in children. Thorax.

[b22-ehp-117-261] Sumer H, Turaclar UT, Onarlioglu T, Ozdemir L, Zwahlen M (2004). The association of biomass fuel combustion on pulmonary function tests in the adult population of Mid-Anatolia. Soz Praventivmed.

[b23-ehp-117-261] Wong TW, Lau TS, Yu TS, Neller A, Wong SL, Tam W (1999). Air pollution and hospital admissions for respiratory and cardiovascular diseases in Hong Kong. Occup Environ Med.

[b24-ehp-117-261] World Health Organization (1975). International Classification of Diseases.

[b25-ehp-117-261] World Health Organization (1997). Health and Environment in Sustainable Development Five Years after the Earth Summit.

[b26-ehp-117-261] Zhou X, Jin Y, He X (1995). A study on the relationship between in-door air pollution and chronic obstructive pulmonary disease in Xuanwei County [in Chinese]. Zhonghua Yu Fang Yi Xue Za Zhi.

